# Neuroregeneration Improved by Sodium-D,L-Beta-Hydroxybutyrate in Primary Neuronal Cultures

**DOI:** 10.3390/ph17091160

**Published:** 2024-08-31

**Authors:** Csilla Ari, Dominic P. D’Agostino, Byeong J. Cha

**Affiliations:** 1 Behavioral Neuroscience Laboratory, Department of Psychology, University of South Florida, Tampa, FL 33620, USA; 2 Ketone Technologies LLC, Tampa, FL 33612, USA; ddagosti@usf.edu; 3 Department of Molecular Pharmacology and Physiology, Morsani College of Medicine, University of South Florida, Tampa, FL 33620, USA; bcha@usf.edu; 4 Institute for Human and Machine Cognition, Ocala, FL 34471, USA

**Keywords:** beta-hydroxybutyrate, synapsin, tubulin, scratch assay, BHB, exogenous ketones, ketone salt, cell migration, neural injury, neuroplasticity

## Abstract

Ketone bodies are considered alternative fuels for the brain when glucose availability is limited. To determine the neuroregenerative potential of D,L-sodium-beta-hydroxybutyrate (D/L-BHB), Sprague Dawley rat primary cortical neurons were exposed to simulated central nervous system injury using a scratch assay. The neuronal cell migration, cell density and degree of regeneration in the damaged areas (gaps) in the absence (control) and presence of BHB (2 mM) were documented with automated live-cell imaging by the CytoSMART system over 24 h, which was followed by immunocytochemistry, labeling synapsin-I and β3-tubulin. The cell density was significantly higher in the gaps with BHB treatment after 24 h compared to the control. In the control, only 1.5% of the measured gap areas became narrower over 24 h, while in the BHB-treated samples 49.23% of the measured gap areas became narrower over 24 h. In the control, the gap expanded by 63.81% post-injury, while the gap size decreased by 10.83% in response to BHB treatment, compared to the baseline. The cell density increased by 97.27% and the gap size was reduced by 74.64% in response to BHB, compared to the control. The distance travelled and velocity of migrating cells were significantly higher with BHB treatment, while more synapsin-I and β3-tubulin were found in the BHB-treated samples after 24 h, compared to the control. The results demonstrate that D/L-BHB enhanced neuronal migration and molecular processes associated with neural regeneration and axonogenesis. These results may have clinical therapeutic applications in the future for nervous system injuries, such as for stroke, concussion and TBI patients.

## 1. Introduction

Neuroregeneration remains one of the significant challenges facing modern medicine. Every year, a significant number of people are affected by nervous system injuries resulting in damage to nervous tissue, or deterioration, leading to the impairment and/or loss of motor functions or certain brain functions, e.g., traumatic brain injury (TBI), Alzheimer’s disease (AD), Parkinson’s disease (PD), concussion, bipolar disorder, post-traumatic stress disorder (PTSD), stroke, fronto-temporal dementia, seizure disorders, etc. Annually, 15 million people worldwide suffer a stroke (WHO 2024), and the estimated global cost of stroke is over USD 721 billion [1]. Globally, the annual incidence of TBI is estimated between 27 and 69 million, while 0.91 million new cases of spinal cord injury and 20.64 million prevalent cases were reported in 2019 [2]. Many TBI survivors live with significant disabilities, resulting in a major socioeconomic burden leading to USD 758 billion in estimated lifetime global economic costs [3]. Such injuries can affect both the peripheral nervous system (PNS) and the central nervous system (CNS), and while the PNS has an intrinsic ability to repair itself, the CNS is incapable of making such a repair. All of these injuries can have long-lasting and devastating consequences with high costs attached to them, while currently, no method exists for repairing and regenerating central nervous system tissue and its underlying components. Accordingly, a potential treatment for brain damage and recovery from brain injury or procedures that cause injuries in the brain tissue (e.g., brain implants) is needed in order to improve patient outcomes. Neuroregenerative treatments that have the potential to improve or treat all types of pathologies ranging from neurodegenerative disorders to CNS trauma are needed. To undergo regrowth or repair and restore the proper functionality of the nervous tissue, ketone bodies can serve as alternative fuel for the brain in scenarios when glucose availability is limited by impaired glucose transport or metabolism. The availability of glucose becomes limited for brain functions when parts of the nervous tissue, neurons, glia, axons, myelin and synapses are damaged. Earlier studies show that neurons become less efficient in using glucose for energy between 6 h and 5 days after acute damage to the nervous system [4]. Neuronal energy can shift towards ketone-derived metabolism following an upregulation of cellular transporters like MCT1 and MCT2 [5]. In addition, several groups have observed a rapid upregulation of ketolytic enzymes (e.g., beta-hydroxybutyrate dehydrogenase) after chemical and ischemic brain insults [6,7]. Although glucose may be the preferred fuel for brain metabolism under normal conditions, in the context of brain injury, ketones can bypass glycolytic restriction to become the predominant fuel. In most neurological disorders, the distinctive characteristic is regional cerebral glucose dysmetabolism/hypometabolism [8]. In fact, neurodegenerative diseases like AD, PD, Huntington’s disease (HD) and epilepsy-related disorders (EDs), as well as neuropsychiatric, chronic mental disorders, such as schizophrenia [9] and major depressive disorder (MDD), have different etiologies; however, they share common metabolic pathogenic manifestations. For example, these conditions are often characterized by glucose hypometabolism linked to impaired insulin signaling, reduced pyruvate dehydrogenase (PDH), the internalization of GLUT-3, mitochondrial dysfunction, oxidative stress, neuroinflammation [10,11,12,13] and amyloidosis [8,14,15,16,17]. Indeed, brain injury, stroke, hypoxia and brain vascular dysfunction are considered risk factors that contribute to the development of glucose-metabolism disorders and induce oxidative stress [8,18], highlighting the need for targeted metabolic-based therapies.

A recent review that analyzed 49 studies showed that the neuroprotective benefits of ketosis in acute central nervous system injury were observed by either inducing endogenous ketosis or by supplying exogenous ketone supplementation [19]. The study highlights that ketosis helped in the reduction in mortality as well as in the reduction in neuronal damage and dysfunction after both brain and spinal cord injury.

Several studies describe the beneficial effects of ketone bodies on brain function, the effect of beta-hydroxybutyrate (BHB) on cognition with type 2 diabetes [20], reducing anxiety in rodents [21] and improving outcome measures in Alzheimer’s patients [22]. BHB is naturally synthetized from acetoacetate in the liver during fasting or during a ketogenic diet (KD). KDs and nutritional ketosis improved impaired cognitive functions [23,24,25,26,27,28] and other neurological conditions, such as anxiety, mood-disturbed behavior, aggression, impaired attention, impaired social functioning, social exploration, decreased alertness and lowered activity levels, in animal models [29,30,31,32,33,34], likely by enhancing the pleiotropic neuroprotective effects. Past and current ketogenic diet studies are composed of hundreds of clinical trials for seizure disorders, neurometabolic diseases, stroke, AD, PD, TBI and psychiatric disorders (bipolar, schizophrenia, anxiety, autism, anorexia, etc.), among other neurological problems like substance use disorders (see clinicaltrials.gov, 20 August 2024).

Our earlier studies showed the neuroprotective potential of ketone supplementation during hyperbaric oxygen exposure and inhalation anesthesia exposure [35,36,37]. Recent studies also show that exogenous ketone supplementation can offer therapeutic potential in psychiatric disorders and aging processes, as well as in age-related neurodegenerative diseases [38,39]. In another study by Yin et al. [40], BHB salts were given subcutaneously over a 30 min period every hour for six hours in rats with a transient middle cerebral artery (MCA) occlusion, simulating an ischemic stroke. The ketone salt (KS) supplementation reduced infarct and penumbral volumes, as shown in MRI scans, and improved the neurological scores in the 24 h after the injury. Later, Tan et al. [41] tested a combination of KD + KS in rats with acute spinal injuries. They found that neuroprotective effects in response to KD + KS were associated with reduced axonal cell death and improved forelimb motor abilities after 4–6 weeks following the injury.

In humans, the current research focuses on the safety, efficacy and tolerability of different formulations of exogenous ketones in people with TBI. In a recent study [42] on 20 patients with stroke, subarachnoid hemorrhage or TBI, it was shown that an enterally administered ketogenic preparation over a 6-day period was well tolerated and sustained ketosis with no negative effect on intracranial and cerebral perfusion pressures. A recent study by Edwards et al. [43] explored diet-induced ketosis with ketone salt supplementation as a potential intervention in PTSD patients and observed the rapid induction and sustainment of ketosis, and patients were compliant while experiencing clinically meaningful improvements in PTSD symptoms.

The role of individual BHB enantiomers is often debated, but D/L-BHB has been shown to have remarkable therapeutic effects for decades [44,45]. In this study, D/L-BHB (also known as R, S-BHB) was used, as recent studies show that, contrary to earlier beliefs, the L-BHB enantiomer has additional neuroprotective signaling properties. While the body produces mostly D-BHB, L-BHB accounts for ~2–3% of endogenous BHB while in a fasted state [46]. Considering the low endogenous production and the fact that the current ketone monitoring devices only measure D-BHB, L-BHB is often overlooked. Thus, after consuming racemic (D/L-BHB) electrolyte salts, the plasma L-BHB levels are elevated [46] but not detectable by current point of care blood ketone meters. However, studies suggest that L-BHB does not only play a role in metabolism, but has functional signaling roles other than its inert function as an intermediate in the beta-oxidation of fats. Studies in rats show that after L-BHB infusion, the molecule can be converted to D-BHB, acetoacetate and acetone (~20%) to be used for energy production [47]. In early studies it was also shown that L-BHB was a favored substrate for the synthesis of sterols and fatty acids for the brain, spinal cord and kidney, but was less favored for oxidation. On the other hand, D-BHB was found to be favored for oxidation, but made less contribution to the synthesis of sterols and fatty acids, suggesting that these isomers are preferentially metabolized in different compartments [48].

Following administration, L-BHB stays elevated in the blood longer than D-BHB, due to its slower metabolism; thus, ketone formulations containing both L- and D-BHB will result in a sustained/prolonged elevation of blood ketones [49]. A sustained elevation of L-BHB may be desirable after a nervous system injury, especially considering L-BHB’s role in epigenetic signaling and the suppression of NLRP3 inflammasome. A previous study demonstrated that, compared to taking D-BHB alone, the racemic mixture led to elevated blood ketone levels that were sustained 4.5× longer (90 min vs. 20 min) [50]. While the L enantiomer stays in the circulation longer than D-BHB [48,51], evidence supports a more important role in signaling [52].

According to a recent study, after the administration of a racemic (D/L-BHB) ketone, salt differences were found in both the uptake and utilization of the two stereoisomers [53]. After the administration of a single, high dose of D/L-BHB, they found that L-BHB increased in all tissues, including in the brain, while D-BHB was increased only in the heart and liver and nearly absent in the brain and muscle tissue. This study highlighted an important aspect of L-BHB supplementation: while L-BHB concentrations increased extensively in the brain, heart, liver and muscle, there was less D-BHB in the heart and liver tissues. For these reasons, and to be consistent with our past work with neuroprotective racemic BHB compounds (esters and salts), we favored the use of sodium D/L-BHB. Since the body naturally makes only a small amount of L-BHB (compared to D-BHB) during endogenously induced ketosis, it may be important to supply an injured nervous system with an L-BHB-containing therapeutic to support neuroregenerative processes.

For our study, we decided to use a D/L-BHB salt, as it may be a safer option for long-term use in contrast to 1,3-butanediol-based molecules. As a di-alcohol with a mild narcotic effect, 1,3-butanediol requires detoxification via the alcohol dehydrogenase pathway, producing a BHB-aldehyde that is potentially not favorable in the context of neuroregeneration. We also avoided the use of unbuffered free BHB acid due to its instability and greater potential to induce acidosis and gastrointestinal intolerance.

The scratch assay, often used as a wound-healing assay, is routinely used as an in vitro economical method for studying cell migration and cellular repair processes. Since brain injury represents a wound, we employed this method as a means to simulate central nervous system injury and monitor associated repair processes and signaling. One of the major advantages of this method is that it generates a strong directional migratory wound-healing response that mimics aspects of cell repair in vivo, thus providing a cell-based model of traumatic brain injury, concussion, stroke or damage associated with brain implants. This standardized method is based on the process by which, when a new artificial gap is created in a cell culture, referred to as a “scratch”, the cells on the edge of the gap will release factors that promote the closure of the “gap”. To determine the rate of cell migration between different treatments, sequential images are taken at regular intervals after injury and during cell migration.

In this study, we investigated rat primary cortical neuron cultures to determine the efficacy and mechanism of D/L-BHB-induced neuroregeneration after simulated injury or trauma. The rationale behind using the ketone body BHB is not only to boost cellular metabolism as an alternative fuel instead of glucose [54], but to enhance pleiotropic neuroregenerative processes. For example, emerging data support the function of BHB in post-translational modification (PTM), in the suppression of inflammation [52] and in favorably influencing endogenous antioxidants and epigenetic regulation [55,56,57].

There are several cell markers that can indicate the functional integrity of the nervous system, such as tubulin and synapsin. Tubulin has an important role in the formation of microtubules, and the proper organization of microtubules has several essential functions, e.g., in mitosis, meiosis, cytoskeletal functions, organellar movement and intracellular transport. Synapsins can be found in the cytoplasmic surface of synaptic vesicles in both CNS and PNS. Synapsin-I, a phosphoprotein, plays an important role in the regulation of axonogenesis and synaptogenesis, as it becomes a substrate of protein kinases. As such, it was desired to monitor the changes in the density of both proteins in the absence and presence of supplemental BHB mimicking therapeutic ketosis.

In this study, the effect of the ketone body D/L-BHB was tested on cell regeneration in rat primary neuronal cell cultures using a scratch assay that was monitored for 24 h using the CytoSMART live cell imaging system. We hypothesized that D/L-BHB treatment would lead to improved neuroregeneration, with a higher density of cell nuclei in the damaged areas, greater cell migration and a higher density of proteins associated with cytoskeletal functions, synaptogenesis and axonogenesis.

## 2. Results

### 2.1. Increased Density of Cell Nuclei and Enhanced Cell Migration with BHB Treatment

**Figure 1:**
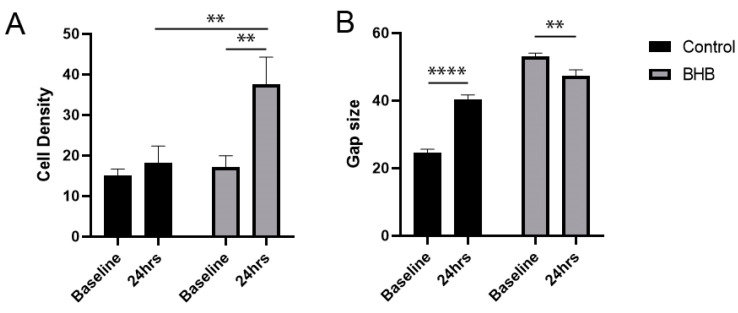
In response to D/L-BHB treatment, there was a significant increase in cell density in the damaged areas after 24 h, compared to the baseline (*p* = 0.001) and compared to the control (*p* = 0.0053); (**A**). The gap size significantly decreased in response to BHB treatment, compared to the baseline (*p* = 0.0019), while the gap size increased in the control (*p* < 0.0001) 24 h after the simulated injury (**B**). ** *p* < 0.01, **** *p* < 0.0001.

**Figure 2:**
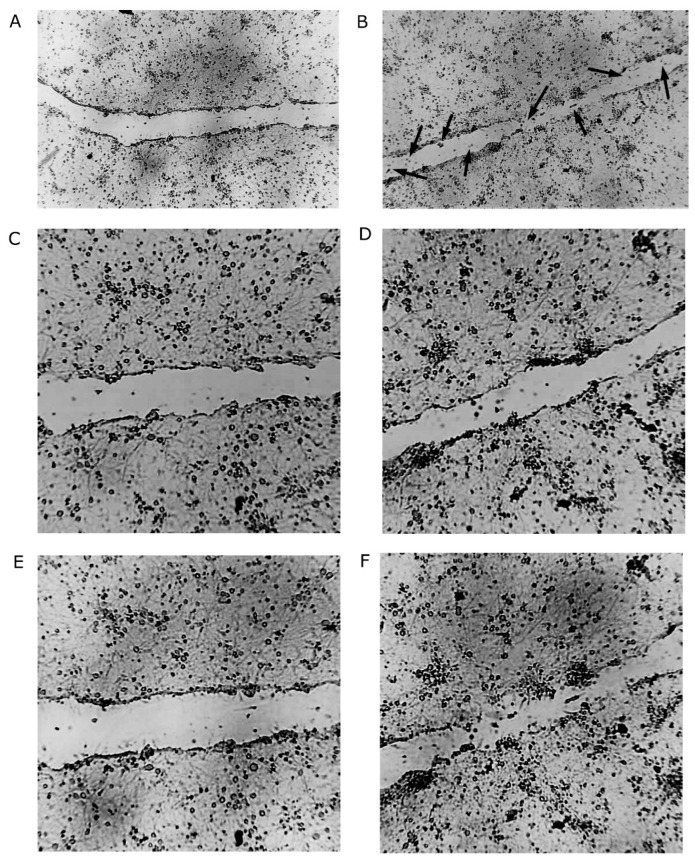
There was a significant increase in cell density in response to D/L-BHB treatment, compared to the control, in the damaged areas 24 h after simulated injury. (**A**) The control group after 24 h: fewer cells were found in the damaged area/gap, compared to the BHB-treated cells. (**B**) The BHB-treated group after 24 h: more cells were found in the damaged area, compared to the control, and the gap of the damaged area almost closed at some areas (arrows). (**C**) The control and (**D**) BHB-treated cells at baseline/at time of injury and (**E**) the control and (**F**) BHB-treated cells 24 h after injury.

**Figure 3:**
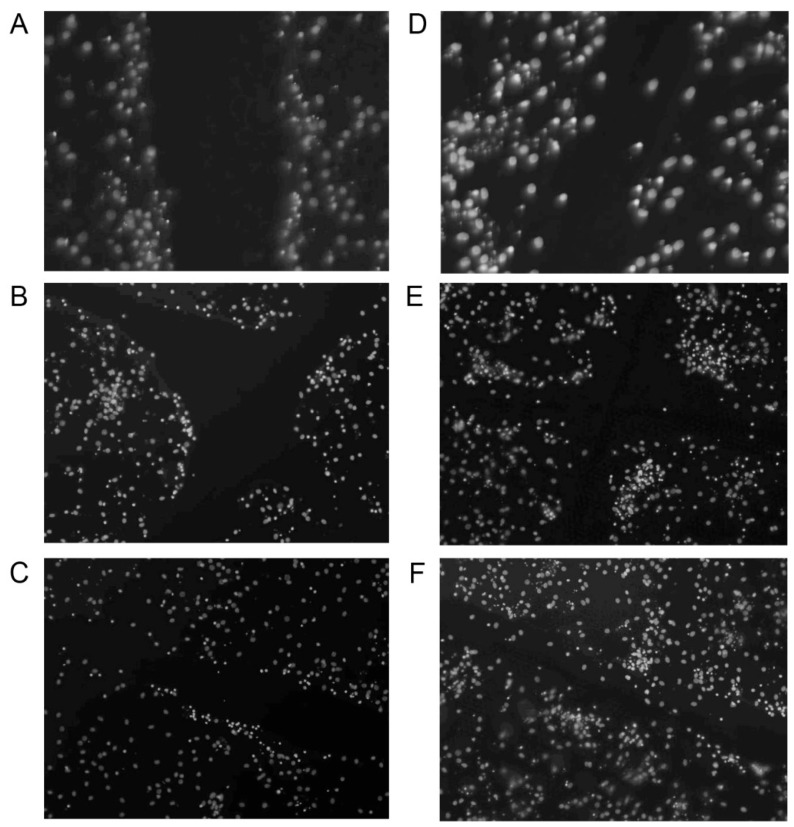
DAPI staining shows the cell density in the damaged areas 24 h after injury. (**A**–**C**) In the control, a lower density of cell nuclei was found in the damaged areas; (**D**–**F**) With the BHB treatment, more DAPI-stained cell nuclei were found in the damaged areas, compared to the control.

**Figure 4:**
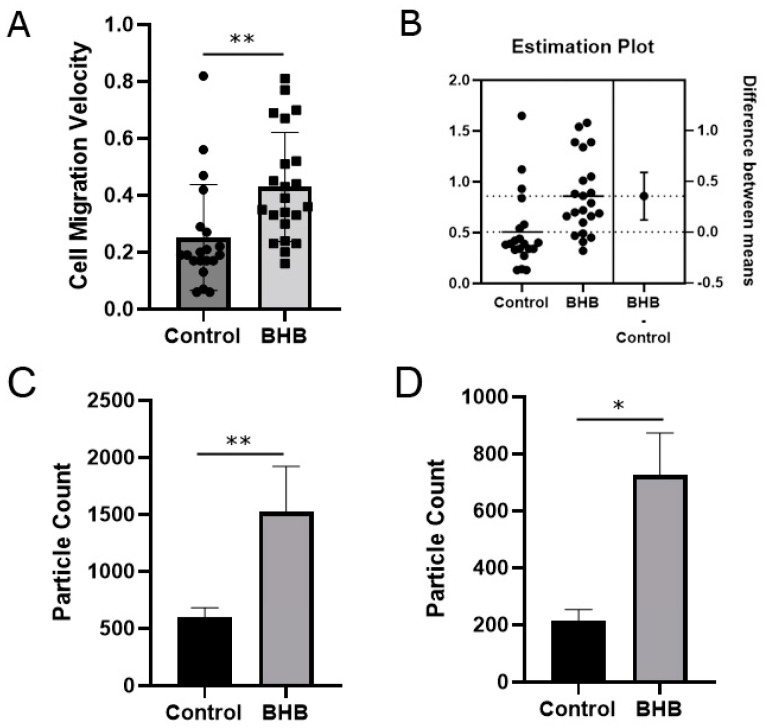
Multiple parameters improved in D/L-BHB-treated samples 24 h after injury. (**A**) The velocity of the cells migrating in the BHB-treated sample was significantly higher (*p* = 0.00041), compared to the control. The graph shows individual values as well as the mean. (**B**) The estimated plot of the distance traveled by cells migrating in the BHB-treated samples were higher, compared to the control (*p* = 0.0041). (**C**) Significantly more synapsin-I was found in the BHB-treated samples, compared to the control (*p* = 0.006). (**D**) Significantly more beta-III-tubulin was found in the BHB-treated samples, compared to the control (*p* = 0.0435). * *p* < 0.05, ** *p* < 0.01.

The cell cultures that were treated with R,S-Sodium-3-hydroxybutyrate (D/L-BHB) showed more intense cell migration, as well as more intense regeneration of the damaged area over the 24 h period (Figure 1, Figure 2, Figure 3 and Figure 4), compared to the control.

In response to BHB treatment, there was a significant increase in cell density in the damaged areas after 24 h, compared to the baseline (*p* = 0.001) and compared to the control (*p* = 0.0053; n = 20; Figure 1A). While the cell density increased by 21.33% in the control, it increased by 118.6% in the BHB-treated samples, compared to their baseline, which represents a 97.27% increase in cell density in response to BHB, compared to the control. The gap size significantly decreased as well in response to BHB treatment, compared to the baseline (*p* = 0.0019), while the gap size increased in the control (*p* < 0.0001) 24 h after the simulated injury (n = 130; Figure 1B). While the gap size increased by 63.81% in the control, it was reduced in the BHB-treated samples by 10.83%, which represents a 74.64% improvement in the BHB-treated samples, compared to the control.

In the control group (Figure 2A,C,E), fewer cells could be observed in the damaged areas/gap after 24 h, compared to BHB-treated cells. The damaged area/gap size increased at 98.5% of the measured areas, while the gap size decreased at 1.5% of the measured areas. In the BHB-treated group (Figure 2B,D,F), more cells could be observed in the damaged areas after 24 h, compared to the control, and the damaged areas/gap almost closed at some places. The gap size increased at 50.77% of the measured areas, while the gap size decreased at 49.23% of the measured areas, which represents a 47.69% improvement in response to the BHB treatment, compared to the control. There was less intense cell migration into the damaged areas in the control (Figure 3A–C), compared to the BHB-treated samples (Figure 3D–F, Supplementary Videos S1 and S2). The velocity of neuron migration in the control samples was 0.252 μm per 15 min, while it was 0.429 μm per 15 min in the BHB-treated samples (*p* = 0.0041, n = 42; Figure 4A), which represents a 70% increase, compared to the control. The estimated distance traveled by neurons in the 15 min intervals was 0.505 μm in the control, while it was 0.859 μm in the BHB-treated samples (*p* = 0.0041, n = 42; Figure 4B).

### 2.2. Increased Synapsin and Tubulin Staining in BHB-Treated Cultures

**Figure 5:**
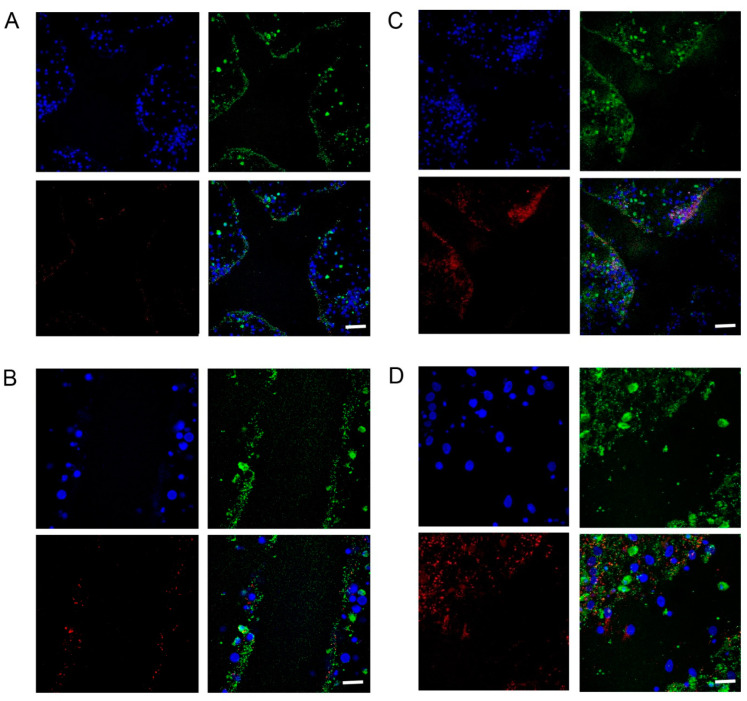
Immunofluorescent staining shows that the density of cell nuclei (blue), synapsin-I (red) and beta-III-tubulin (green) increased with D/L-BHB treatment 24 h after injury. (**A**,**B**) In the control, fewer cell nuclei were visible around the regeneration site, and there was a lower density of synapsin-I and beta-III-tubulin. (**C**,**D**) With the BHB treatment, more cell nuclei were visible around the regeneration site, and there was a higher density of synapsin-I and beta-III-tubulin, compared to the control. Scale bar is 100 μm (**A**,**C**); 50 μm (**B**,**D**).

Fluorescent staining showed significantly more synapsin-I (*p* = 0.006, n = 14; Figure 4C) and significantly more β3-tubulin in the BHB-treated samples (*p* = 0.0435, n = 14; Figure 5C,D), compared to the control (Figure 4D and Figure 5A,B).

Not only the density of synapsin-I (red) and beta-III-tubulin (green), but also the density of cell nuclei (blue) increased with the BHB treatment (Figure 5). In the control group, a smaller number of cell nuclei were visible at the injury/gap/regeneration site (Figure 5A,B), while in the BHB-treated cultures, an increased number of cell nuclei were visible at the injury/gap/regeneration site, along with a higher density of synapsin-I and beta-III-tubulin (Figure 5C,D), when compared to the control.

## 3. Discussion

In this study, our results demonstrate that BHB supplementation in rodent primary neuron cell cultures resulted in an enhanced migration of cells and improved regeneration processes, as more neurons migrated into the damaged areas over 24 h, as compared to the control. Markers that are associated with cytoskeletal functions, synaptogenesis and axonogenesis were also present in a higher density at the damaged areas with D/L-BHB treatment. In this study, we describe the effect of BHB on synapsin-I and beta-III-tubulin for the first time. The presented results illustrate the potential applications of ketogenic compounds containing D/L-BHB for enhancing neuronal regeneration, e.g., after TBI, stroke, concussion or other injury to the CNS.

While these results are preliminary, the potential clinical applications of these findings are broad and may help those diagnosed with brain injury, such as TBI, diffuse axonal injury, concussion, open head injury, closed head injury, Locked in Syndrome, Shaken Baby Syndrome, a penetrating injury following brain implant procedures and more. The common purpose of brain implants is establishing a biomedical prosthesis for brain areas that became dysfunctional after head injuries or stroke. Neural implants for brain stimulations are becoming more common for patients with Parkinson’s disease or clinical depression, while there is also an increasing interest in brain implants for creating brain computer interfaces as well (e.g., NeuraLink). Inducing and enhancing neuroregeneration by D/L-BHB-containing agents administered either orally or intravenously may also be potentially beneficial following an anoxic or hypoxic brain injury, when there is an inadequate supply of oxygen to the brain, e.g., during cerebral infarction, hypoxic ischemic encephalopathy, cerebral ischemia or cerebral hypoxia [58,59].

Nutritional ketosis, when the blood ketone level is 0.5 to 3 mg/dL, can be achieved via carbohydrate restriction or fasting to accelerate the production of ketones and stabilize blood sugar. By suppressing postprandial insulin release, dietary ketosis promotes a metabolic shift toward lipid oxidation and ketone metabolism [60]. When the insulin level remains low, the resulting elevation of lipolysis via hormone-sensitive lipase increases beta-oxidation in the mitochondria of the liver to augment acetyl CoA and ketone body production. This state of nutritional ketosis is normal [61] and is in contrast to, and should not be confused with, the pathophysiologic state of type 1 diabetic ketoacidosis (DKA), a condition associated with pathological hyperketonemia and hyperglycemia. The metabolic derangement associated with DKA is linked exclusively to insulin insufficiency. With exogenous BHB supplementation, the optimal ketone level can be adjusted relatively quickly to maintain the desired therapeutic levels long-term. More importantly, there is no need for severe dietary restriction, which can limit compliance in patients with brain injury or in patients who are unwilling or unable to maintain a restrictive dietary pattern. Similarly, this can be a limitation for the elderly managing the effects of AD, PD and other neurological diseases. Studies suggest that exogenous ketone supplementation was found to be optimal when blood ketone levels were elevated to around 2 mM [62,63], as higher blood ketone levels may increase the potential for acidosis and may trigger a counter-regulatory hormone response (e.g., elevated insulin); therefore, we used a 2 mM concentration based on the rationale that this likely represents a safe and feasible level of ketosis. The safety and efficacy of long-term nutritional ketosis has been documented in multiple randomized controlled trials (RCTs) in epilepsy, and exogenous ketone salt supplements have been on the market for over a decade without FDA reports of serious side effects (some formulations may cause dose-dependent GI symptoms). In addition, from early studies [45] over the last 10 years, there has been a surge of new registered clinical trials with exogenous ketones (clinicaltrials.gov; 20 August 2024), supporting the potential rapid translation of this therapy.

**Figure 6:**
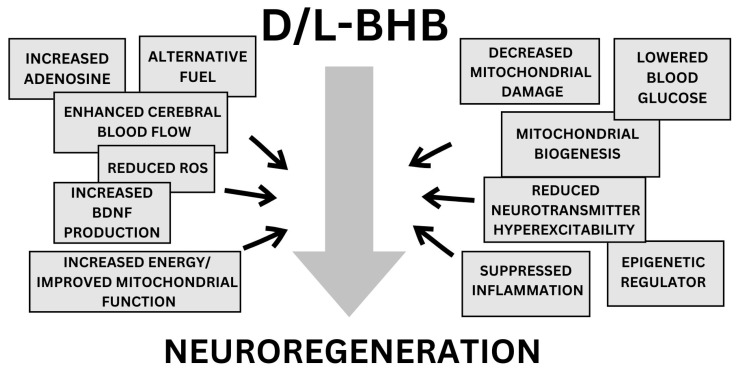
Summary of potential mechanisms that may contribute to the effect of D/L-BHB on neuroregeneration.

Further research is needed in order to identify the exact mechanism of action by which BHB improves neuroregeneration, but there are several potential mechanisms by which the BHB molecule may contribute to the enhancement of neuroregenerative processes. These mechanisms may include, but are not limited to, (1) serving as an alternative fuel source to glucose, (2) enhancing cerebral blood flow, (3) increasing BDNF production, (4) enhancing mitochondrial function, (5) reducing inflammation, (6) reducing oxidative stress, (7) reducing neurotransmitter hyperexcitability, (8) epigenetic regulation, (9) lowering blood glucose and (10) increasing adenosine (Figure 6).

### 3.1. Ketones as Alternative Fuel

During neuronal injury or in people with neurodegenerative diseases, the brain’s ability to use glucose decreases, and thus, in these scenarios, ketone bodies may help to preserve brain energy metabolism. As the density of glucose receptors is reduced by age, the glucose utilization decreases, such as in AD, when neurons gradually lose access to glucose. Glucose hypometabolism is indeed considered a hallmark characteristic of AD [64]. However, in most cases, the brain cells are still able to use ketones for energy, as the monocarboxylic acid transporters (MCT) and the ketolytic enzymes are not impaired, which can lead to a metabolic advantage for the cells that are able to use ketones in addition to or instead of glucose. Providing ketone bodies, as a glucose substitute, has been shown to restore metabolic balance in neurons that are glucose-compromised and subsequently improve cognitive function in type 2 diabetes patients, where cognitive impairment is largely associated with cerebral glucose hypometabolism. Indeed, ketone infusion (i.e., BHB salts) specifically improved working memory performance in patients with type 2 diabetes [20]. In another study, the elevation of plasma BHB concentrations during exhausting exercise using an exogenous ketone supplement led to an attenuated decline in executive function after the exercise, suggesting that consuming the ketone supplement had a cognitive benefit [65].

In order to protect the aging brain, both dietary interventions and administering exogenous ketones showed promising results in a recent study, where the destabilization of the brain network was measured and was found to be correlated with decreased brain activity, as well as decreased cognitive acuity, effects that were found starting at 47 years of age [66]. However, brain network stability was greater following acutely induced ketosis, suggesting that brain network destabilization may be an early sign of hypometabolism, which is associated with dementia and mild cognitive impairment.

Additional evidence supporting the functional role of ketones in brain metabolism and neuroregeneration was highlighted in a recent review analyzing 49 peer-reviewed publications, which showed that ketosis significantly reduced mortality after acute CNS damage, compared to glucose-based interventions. These results support the potential for a ketone-induced reduction in neuronal damage and neuronal dysfunction [19].

### 3.2. Enhanced Cerebral Blood Flow

In addition to the energetic function, the ketone-induced increase in cerebral blood flow may also be responsible for influencing the cerebral metabolic rate, but this relationship has not been firmly established [58,67]. Thus, another potential mechanism by which the ketone body BHB contributes to neuroprotective effects might be through enhancing cerebral blood flow. In a study on nine healthy human subjects, 3-hydroxybutyrate infusions resulted in a cerebral blood flow (CBF) increase of 30% detected by a PET scan. Importantly, the increase in cerebral blood flow was detected in all measured brain areas [58], which potentially may help in removing clotted blood from the brain due to increased CBF. Increased CBF was not affecting the overall metabolic activity and brain pH during acute hyperketonemia [68], so the mechanism by which ketone bodies are mediating this effect is unknown.

### 3.3. Increased BDNF Production

It is known that the BDNF response to exercise can alleviate mental health disorders, such as depression and anxiety, in animal models and in humans [69]; however, some studies show that ketone bodies might also positively affect brain function through increasing BDNF. Earlier studies in both rodents and humans showed a positive correlation between blood concentrations of BHB and BDNF, suggesting that elevated ketone levels may improve cognition, at least in part, through a mechanism that is mediated by BDNF [70,71]. Unfortunately, while there are only a few human studies, this effect seems to be mainly dependent on circulating BHB concentrations [67,72,73]. Circulating ketones may be increased through exogenous ketone supplementation, together with or instead of following the KD, which can rapidly and efficiently increase blood BHB [46,51].

BDNF can affect the proliferation and differentiation of neural precursor cells (NPC) from the hippocampus from where new neurons and glia can be created, therefore playing important roles during the recovery from an injury and aiding in neuroregeneration. Both exogenous ketone supplementation and chronic KD have also been shown to increase BDNF [72,73], but more studies are needed to better understand the effects of different ketone supplements, the effect of combining KD with exogenous ketones and its effect on brain function.

### 3.4. Improved Mitochondrial Function

In patients with head injuries, mitochondrial function was found to be impaired, with a subsequent decrease in ATP production [74] (Verweij et al., 2020). Cahill [75] showed that during fasting, water-soluble ketone bodies can supply up to 60% of the energy requirement of the brain, while in addition to being an energy source, ketone bodies can also provide substrates for anabolism, e.g., for the synthesis of lipids, such as cholesterol in the myelin, which might directly contribute to improving the regeneration of neuronal processes. Ketone bodies, unlike glucose, can bypass the glycolysis in the cytoplasm and can directly enter the citric acid cycle as acetyl-CoA. The generation of energy from ketone bodies takes place in the citric acid cycle and during oxidative phosphorylation, both requiring healthy mitochondrial function. The human brain takes up only 2% of the volume of the body, but it can consume over 20% of its energy [76,77]. Therefore, it presents a very important advantage: BHB molecules, as a fuel source, increase the Gibbs free energy change for ATP by 27%, as compared to glucose, generating 27% more energy for the cells [78].

### 3.5. Suppressed Inflammation

A previous study on cell cultures showed that both L- and D-BHB bind to the hydroxycarboxylic acid receptor 2 (HCAR2) [55], which is expressed in our fat cells, neutrophils, macrophages and other brain cells. The activation of this receptor leads to multiple events that dampen the inflammatory response by inhibiting the production of cytokines. Ketones may suppress inflammation, partly through this mechanism. L-BHB interacts with the immune system and can also lower inflammation by inhibiting the activation of the NLRP3 inflammasome [52]. In that study, the anti-inflammatory effect happened in the presence of both D-BHB and L-BHB, which can have important implications when we consider that L-BHB stays in the bloodstream for longer; therefore, it might have more potential to suppress chronic systemic inflammation or neuroinflammation, which are often associated with CNS injuries. 

### 3.6. Reduced ROS and Decreased Neurotransmitter Hyperexcitability

In TBI, oxidative stress and the increased production of ROS play pivotal roles in the genesis of the delayed harmful effects contributing to permanent damage [79]. Previous studies showed that an increased level of ROS can generate protective and homeostatic processes (e.g., on cellular processes that limit the lifespan, by ROS-dependent and protective pathways); however, during aging, or over a certain level of ROS, it can evoke damages. Based on earlier studies, it is known that BHB and ketogenic diets can protect brain cells against excitotoxicity, as well as oxidative stress during epilepsy and other neurodegenerative diseases; however, the underlying mechanisms are not clear [80].

It has been described that after TBI, there are dynamic changes in the excitatory–inhibitory balance, which is a result of increased glutamate release, faulty reuptake and changes in the receptors and inhibitory interneurons. Therefore, this increase in extracellular glutamate leading to glutamate/GABA imbalance following TBI can contribute to the pathophysiology and neurological dysfunction seen in TBI [81]. It is believed that BHB can affect the production of GABA by increasing the synthesis while pushing the fate of glutamate toward GABA, away from aspartate [82]. Therapeutic ketosis may also increase GABA levels by preserving GABAergic interneurons, elevating GABA accumulation in presynaptic vesicles and increasing GABAA receptor activity [83,84,85,86] while also increasing adenosine levels [87]. It is believed that BHB increases the content of glutamate and decreases that of aspartate in synaptosomes [83]. BHB catabolism was also shown to increase the capacity of GABAergic neurons generating GABA from glutamate [88]. BHB can also inhibit vesicular glutamate transporters, decrease the loading of glutamate to presynaptic vesicles and decrease the release of glutamate, processes that together suppress glutamate-induced neuronal excitability and toxicity [84,89,90,91]. With these processes, BHB can change the inhibitory/excitatory (GABA/glutamate) balance towards inhibition (stabilization), which may contribute to the neuroprotective properties. Another study also suggested that the ketosis/BHB-evoked decrease in the NADP+:NADPH ratio (increased NADPH levels) may increase the levels of dopamine, norepinephrine, epinephrine, serotonin and melatonin. It is important to take into consideration when considering BHB precursors to serve as potential therapeutics for neuroprotection or neuroregeneration that the L-BHB enantiomer has a more potent antiepileptic effect and is also a stronger GABA_B_ receptor agonist [92].

### 3.7. Epigenetic Regulator

Another potential mechanism by which ketones can enhance neuroregeneration is upregulating the transcription of neuroprotective genes that increase the mitochondrial density and endogenous antioxidants [93,94]. Additionally, ketones are capable of inducing neuroprotective epigenetic regulation via β-hydroxybutyrylation and histone deacetylation inhibition [55,57,95,96].

### 3.8. Lower Blood Glucose

Persistent hyperglycemia in severe TBI was an independent predictor of outcomes [97]; therefore, stabilizing and reducing blood sugar is crucial for patients with neuronal injury. Ketogenic supplementation was shown to reduce blood sugar levels in multiple animal models [98], suggesting that elevating blood BHB during the recovery period would reduce the deleterious effects of hyperglycemia on the injured brain.

### 3.9. Increased Adenosine

Increased adenosine A_1_ receptor activity can enhance neuronal survival, supporting it as a therapeutic target for epilepsy and stroke [99,100,101]. Previous studies suggest that this purinergic mechanism leads to the anticonvulsant and neuroprotective effects of ketogenic diets [101]. Ketogenic diets reduce epileptic seizures [102], and the associated augmentation of bioenergetics (ATP) and adenosine levels are associated with this neuroprotective effect [101]. Indeed, adenosine has been shown to offer neuroprotection [103] in models of ischemia, traumatic brain injury [104] and insulin-induced hypoglycemia [105]. Glial cells, specifically astrocytes, regulate purine signaling; therefore, it is likely that the astrocytic network might be critical for abnormal purine signaling after nervous system injury [100,106].

Monocarboxylic acid transporters (MCTs) play an important role for BHB to cross the blood–brain barrier (BBB), since ketone bodies can enter brain cells not only by diffusion, but also by carrier-mediated processes (such as MCTs). Ketone body metabolism in the brain depends not only on its concentration in the blood circulation, but also on the transport across the BBB and into the cells, as well as on the activity of ketone body metabolizing enzymes [107]. Therefore, it is possible that higher concentrations of ketones, the upregulation of transporter proteins and increased enzyme activity would result in an even faster improvement in neuroregenerative processes after nervous system injury, which approaches need to be studied further.

Neural precursor cells (NPC) in the adult brain can transform into new neurons as well as glia cells and can play an important role in recovery from injury. It has been shown that dietary restriction, maybe through the increased production of endogenous ketone bodies, stimulates the expression of neurotrophins and enhances neurogenesis, which has important implications for neuroplasticity and responses of the brain to injury and disease.

Injuries to the CNS are known to have poor prognoses because of their inability to regenerate neurons, in contrast to PNS injuries [108], as CNS axons lose their regenerative ability during development; however, there have been promising directions in this field. Neuroregeneration in the CNS, to synthesize new neurons and connections, is typically inhibited by the extracellular environment and intrinsic factors of neurons. Another reason why CNS neurons typically do not regenerate well is that the axons do not contain all the molecules that are present in cell bodies, such as integrins and growth factor receptors; therefore, without the necessary molecules, the regenerative processes are impaired. It is currently unknown whether BHB contributes to the transport of essential molecules to the axons for enhancing neuroregeneration, but increased tubulin levels show that axonogenesis was likely enhanced in response to BHB treatment. A previous study suggested that the inability of adult neurons to regenerate may be linked to neuronal genes: PTEN and SOCS3 are proteins that may inactivate regeneration in CNS neurons by inhibiting AKT and JAK/STAT signaling, respectively. It was found that increasing mTOR activity via the deletion of PTEN and SOCS3 can enhance axonal regrowth [109,110]. Interestingly, the KD has been shown to inhibit the mTOR signaling pathway [111], while BHB may increase mTOR activation after exercise [112]. Another process that prevents CNS neuroregeneration is impaired transport mechanisms to the site of injury. Indeed, glial scars or the lack of myelin clearance by macrophages and microglia can prevent neuronal regeneration by forming a chemical and physical barrier to axonal extension [113]. Reducing glial scars and inhibitory signals in the extracellular matrix has been suggested to be a promising direction in enhancing neuroregeneration; however, in our experiments, we were not able to study the impact of either of these factors. Chondroitinase ABC showed a promising approach for axonal regeneration in spinal cord injuries [114] when combined with Schwann cell transplants [115] or peripheral nerve autografts [116]. The depletion of keratan sulfate proteoglycans (KSPGs) was also found to suppress the inhibition of nerve regeneration [56].

Neuroplasticity in the nervous system optimizes neural networks after brain injury, and the damaged cortex is remapped to another part of the cortex [117]. It was suggested that the alteration of the GABAergic interneuron circuitry may contribute to cortical remapping, as well as to the growth of new connections [118] and to the generation of action potential [119,120]. Other studies show great potential for neuroregeneration when rehabilitation is combined with serotonin, dopamine and neuromodulation to induce plasticity [121,122]. Indeed, BHB is known to regulate neurotransmitter balance and has been shown to increase GABA; therefore, it may contribute to enhancing the cortical remapping after brain injury. Neurotrophic factors, such as BDNF, neurotrophin-3 (NT-3) and nerve growth factor (NGF), all influence the regeneration of the nervous system, and using these molecules led to recovery in rodents [123] and primates [124]. Reducing inflammation was also identified as a key part of treatments for neuroregeneration [125]. BHB has also been shown to increase BDNF and reduce inflammation; therefore, this molecule may have great potential to enhance neuroplasticity and neural regeneration after CNS injuries [126].

In the recent review by Gambardella et al. [19], they show that the benefit of ketosis in the 49 reviewed studies was independent of the timing (i.e., precurrent vs. concurrent to neurological insult) and whether the ketosis was induced endogenously or exogenously, but the BHB level was a predictor of the effect size of neuroprotection. Some of the limitations of the present study include that the ketone intervention was introduced only concurrently and only one concentration of BHB treatment was tested. In order to have a better understanding and to further improve neuroregenerative processes, we need future dose response studies and a pre-treatment of the BHB intervention. Our study was performed on cell cultures from rodents; therefore, caution is warranted in extrapolating the results, although murine animal models are proven to be a satisfactory translational model widely used in neuroscience [127], as their CNS has many similarities with human CNS [128]. Another limitation of our study was that primary neuronal cultures that lack cellular heterogeneity normally present in the brain (e.g., astroglia, microglia, endothelial cells) were used; therefore, these cultures do not precisely mimic the microenvironment present in the brain. In addition, the cerebrospinal fluid, capillaries and barriers are not present in these cultures. Besides primary cultures, immortalized cell lines can also be used for similar experiments, such as neuronal progenitor cells or stem cell cultures, but the use of these cells can be restricted due to ethical problems [129], e.g., adult stem cell extraction can be difficult or not feasible [130]. More recently, 3D tissue models have also gotten popular in representing the three-dimensional complexity of the organs, which may be the next possible direction for continuing these studies.

The current study only measured changes during the acute phase of 24 h; however, continued chronic treatment with either intravenous or oral BHB administration may offer additional benefits and may reveal changes in growth patterns, regeneration processes and associated signaling. While keeping blood ketone levels elevated by using a high-fat diet, e.g., a ketogenic diet, for longer periods of time can be achieved in some clinical scenarios, the length of time it takes to achieve elevated blood ketone levels may take days, and a lack of compliance and dyslipidemia may be problematic for many patients. Therefore, exogenous ketone supplementation represents a more feasible and faster alternative that can be used with or without diet therapy. Oral BHB supplementation can be dose-adjusted to achieve almost immediate therapeutic ketosis and is more manageable to sustain at a stable level and to adhere to in the long term, which are all critical aspects when considering it for therapeutic applicability, especially for patients with brain injuries.

## 4. Materials and Methods

Cortical neurons of embryonic rats (Sprague Dawley, E18) were cultured. The cortical neurons were purchased from ThermoFisher Scientific (Waltham, MA, USA) in a vial as E18 neurons. The primary rat cortex neurons are isolated and cryopreserved from day-18 rat embryos. This is a flexible, ready-to-use and standardized alternative to fresh primary dissociated neuronal cultures; each vial contains 1 million cells per mL. These neurons consistently yield post-thaw viability in the range of 50–80% and have high neuronal purity. The cells were suspended in DMEM supplemented with fetal bovine serum (FBS, 10%, heat-inactivated), penicillin (100 IU/mL), streptomycin (100 mg/mL) and amphotericin B1 (0.25 mg/mL) (Antibiotic/Antimycotic) and plated on poly-L-lysine-coated coverslips. DMEM was used as a basal medium with no glucose and glutamine. After 24 h of incubation, the DMEM solution was replaced with Neurobasal media supplemented with B-27 (10 mL) and 0.5 mM L-glutamine. The cells were plated in Neurobasal medium supplemented with B-27 (control medium) on poly-L-lysine-coated (4.5 μg/cm^2^) coverslips in six-well plates by seeding at 2 × 10^5^ live cells per 1 mL of the medium. The scratch assay was performed on 3-week-old neuronal cultures, and ~200–300-μm wide straight lines were scratched in the center of each culture (crossing in the middle at a 90-degree angle to provide a reference point) as a simulated injury using a sterile 200 μL micropipette tip. The cultures were exposed to either regular media (control) or regular media supplemented with 2 mM R,S-Sodium-3-hydroxybutyrate (D,L-BHB, Sigma, Burlington, VT, USA) for 24 h after performing the simulated injury.

### 4.1. Immunocytochemistry

Twenty-four hours after performing the simulated injury/scratch assay, the cultured neurons were fixed with methanol, permeabilized with 0.3% Triton X in 0.1% PBS for 5 min at room temperature and rinsed three times with PBS. The cultures were blocked with 10% normal goat serum (NGS) for 60 min at room temperature and then exposed to primary antibodies in 0.3% Triton X with 0.1% PBS overnight at 4 °C. The following primary antibodies were used, diluted in 5% goat serum solution: rabbit polyclonal anti-Synapsin I (1 μg/mL, Abcam, Cambridge, UK) and mouse monoclonal anti-β3-tubulin (1 μg/mL, Abcam). After exposure to primary antibodies, the cultures were rinsed three times with PBS and further incubated at room temperature for 60 min with an AlexaFluor^®^488 Goat anti-Mouse secondary antibody (Thermofisher Scientific, Waltham, MA, USA) diluted at 2 μg/mL (shown in green) and AlexaFluor^®^594 Goat anti-Rabbit secondary antibody at 2 μg/mL (shown in red). The cells were rinsed three times with PBS before staining the nuclear DNA with DAPI (3 ng/mL, Thermofisher Scientific, Waltham, MA, USA) for 10 min (shown in blue). The process was finished by rinsing once with PBS.

### 4.2. Imaging

The images were taken every 15 min to monitor the cell regeneration and cell migration into the damaged areas over 24 h by the CytoSMART© system (Version1), starting after the simulated injury/scratch assay was performed. A timelapse video was created using the series of still images over 24 h. Fluorescence imaging was performed using an Olympus FV1200 Laser Scanning Confocal Microscope (Olympus, Tokyo, Japan) to visualize DAPI, β3-tubulin and synapsin-I. The images were analyzed, and changes in cell density and changes in the width of the damaged area (gap size) were measured using the ImageJ/FIJI (1.54 d) program. The distance travelled and velocity of cell migration were measured in micrometers based on the time points when the photos were taken, but the migration intensity of the cells were not known between those time points. For the visualization and quantification of the immunocytochemical reactions, the entire volume of a Z-stack of the sample region of interest (ROI) was collected to avoid bias for selecting a specific focal plane, using ImageJ/FIJI (1.54 d). The fluorescent signal acquisition setting was kept the same within an experiment so that the signals could be quantitated and compared between treatment groups. The specimens were labeled with three probes, Alexa Fluor 594 (A594), A488 and DAPI. The DAPI, Alexa 488 and Alexa 594 were excited by 405 nm, 488 nm and 559 nm lasers, and the corresponding emission signals (425~475 nm for DAPI, 530~570 nm for Alexa 488 and 565~660 nm for Alexa 559) were acquired sequentially to avoid bleed-thorough between the emission channels. The image analysis program ImageJ/FIJI was used to threshold the positive regions (which appeared as particles) of each staining and, subsequently, to measure the number of particles. The threshold parameters were kept the same for all the images being compared. There were two platings and 12 samples per treatment per plating. The number of ROIs were used as an n sample size.

### 4.3. Statistics

All data were presented as the mean ± standard error of the mean (S.E.M.). GraphPad Prism version 9.2.0 was used for the data analysis. Significance was tested using the two-tailed Student’s *t*-test and two-way ANOVA. The results were considered significant when *p* < 0.05.

## 5. Conclusions

In conclusion, the presented results suggest that exogenous ketone supplementation containing sodium-D/L-BHB augments cellular and molecular neuroregenerative processes after simulated CNS injury. Considering that starting a ketogenic diet approach after a brain injury would require days to achieve elevated blood BHB levels, exogenous BHB supplementation in commercially available supplements can result in an almost immediate blood BHB elevation, which represents a promising potential future application for enhancing neuroregeneration after brain or spinal cord injuries. These molecules may have clinical relevance—for example, for injuries caused by stroke, TBI, skull fractures, concussion and sub-concussive events, which may be responsive to ketone therapy, and the efficacy of conventional post-stroke and post-TBI rehabilitation therapies or the recovery after brain implants may be enhanced by exogenous ketone supplementation containing sodium-D/L-BHB. With further research these findings may translate into clinical treatments, but human clinical trials are needed using BHB supplementation for patients with neuronal injuries. While adhering to a high-fat ketogenic diet may be challenging for many, especially pediatric and elderly patients, the exogenous BHB supplementation may present a viable alternative. We hope that these results will inspire further studies in this direction and may help overcome regulatory challenges that currently prevent clinical applicability. This metabolic-based therapy may enhance other treatments, but more research is needed regarding these specific applications and combination therapies in humans. While the preliminary studies are promising, further research is needed to optimize the formulations, dose, timing and scheduling of therapeutic ketone administration and to explore whether ketone supplementation with D/L-BHB can be potentially additive or synergistic with other existing, dietary or pharmacological therapies for nervous system injuries.

## Data Availability

The original contributions presented in the study are included in the article/Supplementary Materials, further inquiries can be directed to the corresponding authors.

## Supplementary Materials

The following supporting information can be downloaded at: https://www.mdpi.com/article/10.3390/ph17091160/s1, Video S1: Timelapse video made from the photographs of a control neuronal culture taken by CytoSMART every 15 min over 24 h. Video S2: Timelapse video made from the photographs of a D/L-BHB-treated neuronal culture taken by CytoSMART every 15 min over 24 h.
